# Chitosan Nanoparticles-Preparation, Characterization and Their Combination with *Ginkgo biloba* Extract in Preliminary In Vitro Studies

**DOI:** 10.3390/molecules28134950

**Published:** 2023-06-23

**Authors:** Monika Owczarek, Lucyna Herczyńska, Przemysław Sitarek, Tomasz Kowalczyk, Ewelina Synowiec, Tomasz Śliwiński, Izabella Krucińska

**Affiliations:** 1 Łukasiewicz Research Network—Lodz Institute of Technology, Skłodowskiej-Curie 19/27, 90-570 Lodz, Poland; 2 Institute of Materials Science of Textiles and Polymer Composites, Faculty of Material Technologies and Textile Design, Lodz University of Technology, Żeromskiego 116, 90-924 Lodz, Poland; lucyna.herczynska@p.lodz.pl; 3 Department of Medical Biology, Medical University of Lodz, ul. Muszyńskiego 1, 90-151 Lodz, Poland; przemyslaw.sitarek@umed.lodz.pl; 4 Department of Molecular Biotechnology and Genetics, Faculty of Biology and Environmental Protection, University of Lodz, Banacha 12/16, 90-237 Lodz, Poland; tomasz.kowalczyk@biol.uni.lodz.pl; 5 Laboratory of Medical Genetics, Faculty of Biology and Environmental Protection, University of Lodz, Pomorska 141/143, 90-236 Lodz, Poland; ewelina.synowiec@biol.uni.lodz.pl; 6 Department of Medical Biochemistry, Medical University of Lodz, 90-001 Lodz, Poland; tomasz.sliwinski@biol.uni.lodz.pl

**Keywords:** chitosan nanoparticles, *Ginkgo biloba* extract, drug release, cytotoxicity

## Abstract

Nanoparticles (NPs), due to their size, have a key position in nanotechnology as a spectrum of solutions in medicine. NPs improve the ability of active substances to penetrate various routes: transdermal, but also digestive (active endocytosis), respiratory and injection. Chitosan, an N-deacetylated derivative of chitin, is a natural biodegradable cationic polymer with antioxidant, anti-inflammatory and antimicrobial properties. Cross-linked chitosan is an excellent matrix for the production of nanoparticles containing active substances, e.g., the *Ginkgo biloba* extract (GBE). Chitosan nanoparticles with the *Ginkgo biloba* extract (GBE) were obtained by ion gelation using TPP as a cross-linking agent. The obtained product was characterized in terms of morphology and size based on SEM and Zeta Sizer analyses as well as an effective encapsulation of GBE in nanoparticles—FTIR-ATR and UV-Vis analyses. The kinetics of release of the active substance in water and physiological saline were checked. Biological studies were carried out on normal and cancer cell lines to check the cytotoxic effect of GBE, chitosan nanoparticles and a combination of the chitosan nanoparticles with GBE. The obtained nanoparticles contained and released GBE encapsulated in research media. Pure NPs, GBE and a combination of NPs and the extract showed cytotoxicity against tumor cells, with no cytotoxicity against the physiological cell line.

## 1. Introduction

*Ginkgo biloba* trees are a relict and endemic species, the only one that survived the entire Cenozoic era [1]. According to the data contained in the literature, the oldest specimens are over 4000 years old, grow in China and their height is about 30 m [2]. Nowadays, there are huge plantations of *Ginkgo biloba* trees in the United States and France due to their therapeutic effect and content of active substances in the leaves (both green and yellow) [1]. Since ancient times, *Ginkgo biloba* leaf extract (GBE) has been used in the prevention and treatment of many diseases due to its minimal side effects. It is used as one of the most commonly consumed dietary supplements that improve memory and concentration [3]. It has also been shown that GBE effectively improves blood microcirculation in the limbs and brain by increasing the supply of nutrients and oxygen, prevents the formation of clots, strengthens capillary walls and protects nerve cells against damage with limited oxygen in the environment [3,4]. GBE is widely used in hypertension as well as inflammation [5,6,7,8]. The GBE leaf extract also has an antioxidative effect—it removes harmful free radicals. In addition, numerous studies have proven its anticancer, anti-mutagenic, anti-asthmatic activity as well as its ability to accelerate the wound-healing process [2,4,5]. Studies have shown that GBE has antiproliferative effects and induces apoptosis in various types of cancer, such as pancreatic, liver and colon [9,10].

GBE is a source of various compounds: flavonoids, terpene lactones, terpenoids, polyphenols, organic acids, carbohydrates, EFAs, inorganic salts, amino acids and others [1,11,12]. As a result of biological studies, it has been shown that its therapeutic activity is caused by the fraction of flavonol glycosides and terpene derivatives [1,12]. According to the standards of the Chinese Pharmacopoeia, the content of flavonoids and terpene lactones in GBE should be equal to or higher than 24% and 6%, respectively [13]. The flavonoid fraction consists of three flavonols: quercetin, kaempferol and isorhamnetin. Quercetin has antioxidant, anti-allergic, anti-viral and anticancer effects [14]. Kaempferol has antioxidant, anti-inflammatory, antimicrobial, cardiovascular, and neuroprotective properties [15]. Isorhamnetin is a flavonoid having anti-tuberculosis, anticancer and anti-inflammatory activity [16]. The terpenoid fraction consists of ginkgolides and bilobalides. Ginkgolides inhibit the platelet-activating factor, reducing aggregability, and bilobalide has an anti-apoptotic effect on nerve cells, and prevents cerebral ischemia, Alzheimer’s and Parkinson’s diseases [17]. An important flavonol glycoside is also rutin, which may constitute ≤ 4% of *Ginkgo biloba* extract [18] and has a wide range of pharmacological properties, including antioxidant, cytoprotective, anticancer, neuro-, vascular- and cardioprotective [19]. Due to the many health benefits of GBE, its addition is widely used, for example, in medicinal products, cosmetics, food additives and functional drinks [5].

Recently, researchers have focused primarily on the development of therapeutically active polymer nanoparticles—NPs (with a size in the range of 1–1000 nm)—in order to improve the ability of active substances to penetrate various routes, including: transdermal, but also digestive (active endocytosis), respiratory and injection [20]. Polymer NPs have the possibility of easier modification of chemical and physical properties, thus reducing the loss of the drug enclosed inside, while increasing the penetration of the drug by absorption and ensuring a controlled release [21]. Encapsulation of plant extract is an effective method that enables long-term storage, protection of the properties of sealed substances and the extension of their release time at the application places [22]. NPs based on various polymers are usually prepared using ionic or covalent cross-linking methods, precipitation methods, polymerization or self-assembly [23]. The matrix of polymer NPs usually consists of natural polymers (chitosan, gelatin, alginate, etc.), synthetic polymers (PLA, PCL, PLGA, cyclodextrin, etc.) and their blends [21,24]. The most frequently studied nanoparticles from natural polymers for dermal applications are NPs based on chitosan [21,25,26]. Chitosan, an N-deacetylated derivative of chitin, is a natural biodegradable cationic polymer with antioxidant, anti-inflammatory and antimicrobial properties. These unique properties make this biopolymer a suitable carrier of therapeutic agents for the treatment of dermatological diseases [25]. Moreover, at physiological pH, the primary –NH_2_ groups of chitosan are protonated, and therefore, chitosan shows a positive charge and can be used to create NPs as a result of cross-linking with polyanions (e.g., pentasodium triphosphate—TPP) for the effective encapsulation of negatively charged drugs [25]. This cationic polymer is biocompatible, has hemostatic properties and can bind with negatively charged active substances due to electrostatic interactions, thus increasing cell bioavailability and internalization [3,21]. Given their stability of and ease of surface modification, NPs are widely used in various industrial sectors, especially medical, pharmaceutical and veterinary [21].

Another important research aspect is focusing on the development of an efficient transdermal system, e.g., in the form of nanoparticles, which will allow active substances to penetrate the deeper layers of the skin with the least possible loss. The largest, multi-layer organ of the human body is the skin, which has an area of 1.5 to 2 m^2^ and accounts for nearly 1/6 of the body weight. The skin performs several important functions in the body. Among others, it constitutes a barrier between the body and the external environment, protects against harmful biological, chemical and mechanical factors, maintains fluid homeostasis in the body and performs active functions: thermoregulation, secretion, excretion, sensory, metabolic regulation and defense [26,27]. The skin consists of three layers: epidermis, dermis and subcutaneous fat [28]. Active substances can penetrate the epidermal layer in two ways—through the stratum corneum (transepidermal transport) and through skin appendages, i.e., sweat glands, sebaceous glands and hair follicles (transfollicular transport). Transepidermal transport to the dermis can take place via the intercellular route (intercellular transport, typical for lipophilic substances, soluble in the intercellular cement) and the transcellular route (through epidermal cells—corneocytes, typical for hydrophilic substances, insoluble in the cell cement) [29,30]. On the other hand, transfollicular transport is a process involving the penetration of active compounds into the dermis along the hair follicles. This type of transport occurs in the case of lipophilic substances that are very soluble in secretions of the sebaceous glands located near the hair follicles [31]. Active substances and NPs that come into direct contact with the skin penetrate it primarily by passive diffusion, which was described by Fick’s first law [32].

The aim of the presented work was to obtain and characterize chitosan nanoparticles with *Ginkgo biloba* extract—Ch(GB)NPs. The rate of release of the core material from nanoparticles was tested in two research media to check which test system allows for the constant and slow release of the active substance over time. In addition, for the first time, biological studies were carried out on various cell lines (Human Gingival Fibroblast—HGF-1; Human Cervical Cancer—HeLa; and Human Ovarian Cancer—PEA1) to check the cytotoxic effect of pure GBE, pure chitosan nanoparticles (ChNPs) and a combination of chitosan nanoparticles with the *Ginkgo biloba* extract, Ch(GB)NPs.

## 2. Results

### 2.1. Characteristic of Ch(GB)NPs

**Figure 1:**
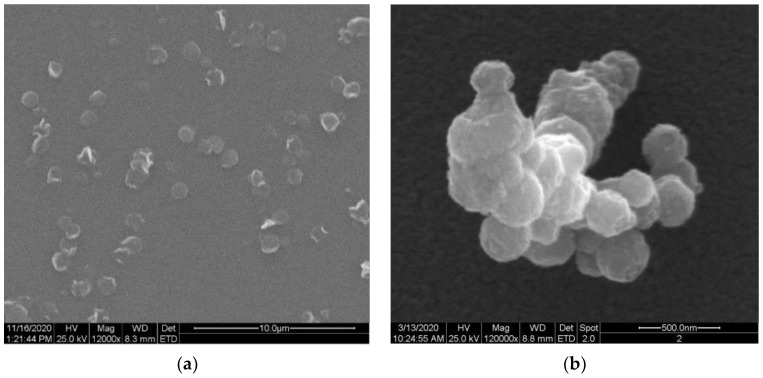
SEM images of Ch(GB)NPs (**a**) at 12,000× magnification and (**b**) at 120,000× magnification.

Chitosan-coated nanoparticles loaded with GBE were obtained by an ionic gelation method as a result of the reaction between positively charged –NH_2_ groups of chitosan and negatively charged groups of the crosslinker (TPP). *Ginkgo biloba* extract was dissolved in a 0.5% chitosan solution, and then TPP was added to allow the active ingredient to be encapsulated. The shape and structure of the surface of Ch(GB)NPs were observed by scanning electron microscopy (SEM; Figure 1). In the SEM images (Figure 1a), it is observed that some of the nanoparticles are quite spherical with a regular edge, while the rest are irregular and with uneven edges. Figure 1b shows Ch(GB)NPs as an aggregate of several spherical and irregular particles with a rather rough surface.

The mean diameter and PDI of the tested nanoparticles in the water medium were observed by the DLS method, and the results are described in Table 1.

**Figure 2:**
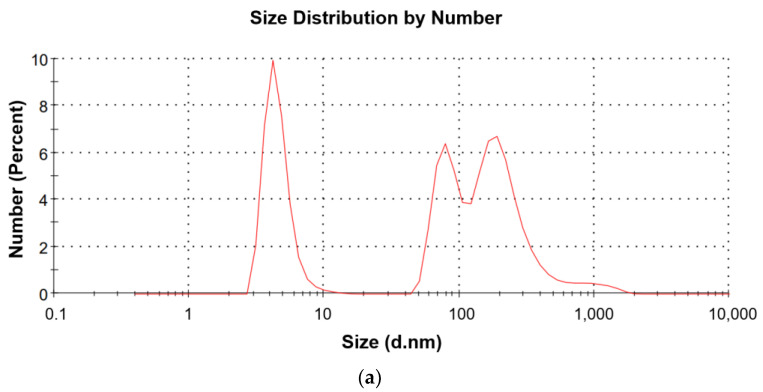

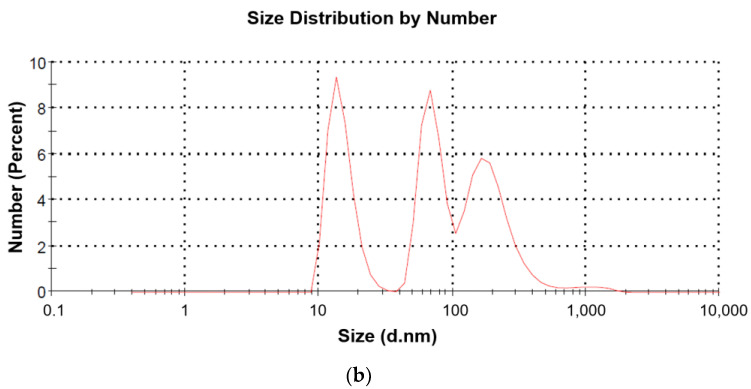
Dependence of the intensity on the size of nanoparticles in the tested Ch(GB)NPs sample, (**a**) without initial sonification and (**b**) with a 10 min initial sonification.

The average diameter of Ch(GB)NPs (without initial sonification) in the tested sample was 454.2 nm. A total of 5 min of sonification allowed to reduce the average diameter of nanoparticles to the level of 374.9 nm, while 10 min of sonification allowed for obtaining nanoparticles with the smallest average diameter of 358.9 nm. Figure 2a shows the most numerous fractions (fractions with an average diameter of 4.6, 86.3 and 269.2 nm) related to the unit measurement of a small sample of the obtained nanoparticles (without preliminary sonification). Figure 2b shows the most numerous fractions of the sample after 10 min of sonification (fractions with an average diameter of 14.87, 71.97 and 202.9 nm).

### 2.2. Encapsulation and Loading of GBE Efficiency (GBE EE%)

Analyzing the concentration of *Ginkgo biloba* extract before and after encapsulation, the average of the extract closure in the chitosan nanoparticles from five measurements was 53.93 ± 1.62%.

### 2.3. Kinetics of Core Material Release

The release of *Ginkgo biloba* extract in water and in saline from the obtained Ch(GB)NPs, at the temperature of 33 °C, was carried out. The concentration of the released core material was determined by UV-Vis spectroscopy, examining the absorption at a wavelength in the range of 257–270 nm, characteristic for rutin.

**Figure 3:**
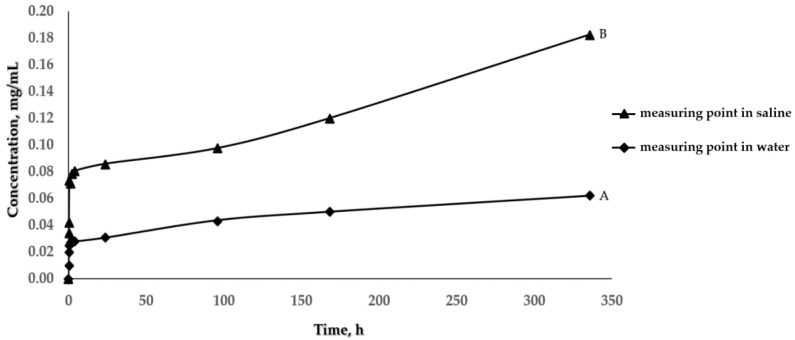
The rate of release of *Ginkgo biloba* extract from Ch(GB)NPs in water, pH 7.0 (**A**) and in saline, pH 5.8 (**B**).

Figure 3 shows the release curves of *Ginkgo biloba* extract from Ch(GB)NPs. Based on the results presented in this figure, it can be concluded that nanoparticles containing *Ginkgo biloba* are characterized by a different release rate of the core material, depending on the medium used. In the first 24 h of the test, the extract is released into the environment quite intensively in both media (the concentration of the released extract in saline is much higher than in the water medium). After 24 h, the amount of released core material changes slightly by stabilizing in the water medium and increasing more slowly in saline. The intensity of the core material releasing is higher for saline.

In order to better understand the drug-release mechanism, the release profiles at pH 7.0 and 5.8 were analyzed by fitting the obtained release data to different kinetic models. The regression coefficient values are summarized in Table 2. The results revealed good fitting profiles with the mathematical models (Supplementary Material).

### 2.4. FT-IR Analysis

**Figure 4:**
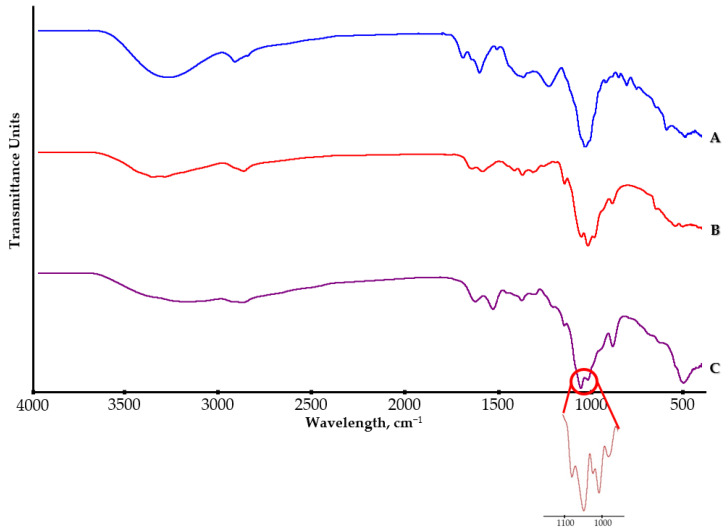
FTIR-ATR spectra for GBE (A), pure chitosan powder (B) and Ch(GB)NPs (C).

As a result of the FTIR-ATR analysis (Figure 4), we obtained spectra for the tested samples (*Ginkgo biloba* extract—GBE, pure chitosan and chitosan nanoparticles with closed *Ginkgo biloba* extract—Ch(GB)NPs).

The spectrum of GBE shows characteristic bands assigned to the H-bonded O–H stretch at the wavelength of 3281 cm^−1^ as well as antisymmetric and symmetric C–H stretches at 2924 cm^−1^ and 2861 cm^−1^. The peaks for the ester carbonyl C=O stretch at 1694 cm^−1^, the aliphatic C–H bond at 1512 and 1370 cm^−1^ as well as the symmetric (1235 cm^−1^) and alcohol C–O stretches (at 1039 cm^−1^) can be observed.

In the spectrum of chitosan at the wavelength of 3354 cm^−1^, the peak characteristic of O–H stretching bonds can be observed. The bands at the wavelength of 2871 cm^−1^ for the investigated polymer are assigned to symmetric and asymmetric C–H bonds. At 1650 cm^−1^, there is a characteristic band stretching the C=O bond in the N-acetyl group (amide I) and at 1589 cm^−1^—a band characteristic for the amino group (amide II). The peaks at 1418 cm^−1^ and 1319 cm^−1^ are characterized by symmetrical deformations of CH_2_ and CH_3_ bonds. The spectrum also contains typical peaks for C–O–C bonds, at 1150 cm^−1^ for a glycosidic bond and at 1059 cm^−1^ for a saccharide ring bond. The band present at 894 cm^−1^ is assigned to the hydroxyl group.

In the case of the FTIR-ATR spectrum for Ch(GB)NPs in relation to the spectra of GBE and pure chitosan, the characteristic peak for the extract encapsulated in chitosan nanoparticles cannot be seen due to the overlapping of several characteristic peaks for all the tested components.

### 2.5. Cytotoxic Effect

**Figure 5:**
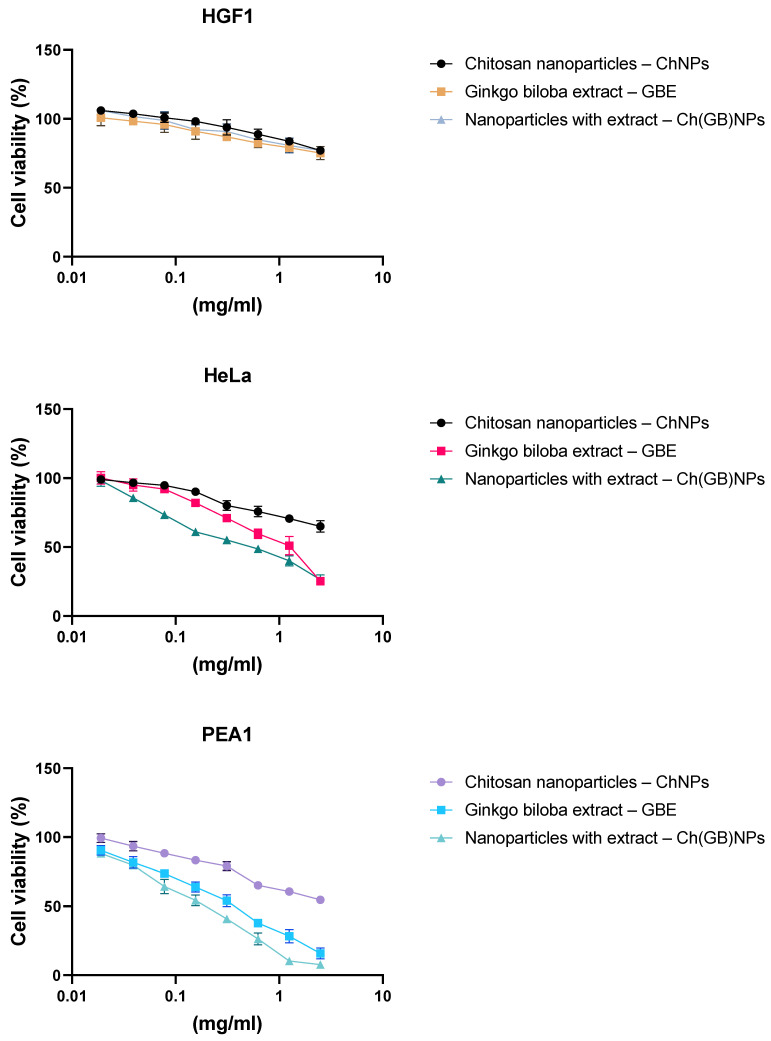
Effects of GBE, ChNPs and Ch(GB)NPs on HGF-1, HeLa and PEA1 cell viability, as determined by the MTT assay after 24 h. Values represent the means ± SD as a percentage (%) of the control.

Our studies showed moderate cytotoxic effects for the normal HGF-1 cell line after treatment with chitosan nanoparticles, the *Ginkgo biloba* extract and a combination of chitosan nanoparticles with the plant extract in the tested concentration range. In turn, all applied combinations showed a cytotoxic effect for the two tested HeLa and PEA1 cancer cell lines. For the HeLa cancer cell line, a weaker cytotoxic effect was demonstrated after treatment with chitosan nanoparticles. IC_50_ for the *Ginkgo biloba* extract was about 0.975 mg/mL, and for the chitosan nanoparticles in combination with the IC_50_ extract, it was about 0.505 mg/mL, which showed a strengthening effect. For the PEA1 cancer cell line, a stronger cytotoxic effect was observed for the extract of *Ginkgo biloba* with IC_50_ at about 0.335 mg/mL, and for the extract with chitosan nanoparticles, it was about 0.178 mg/mL. In both cases, a strengthening effect was observed for the extract with chitosan nanoparticles (Figure 5).

## 3. Discussion

In this research, chitosan nanoparticles containing GBE with an average diameter of 454.2 nm were obtained in the ion gelation process. Ch(GB)NPs were characterized in terms of size and morphology, as well as shape and surface by SEM and Zeta Sizer analyses. The obtained nanoparticle fractions in terms of size correspond to those nanoparticles that are used to administer active substances through the skin. Patzelt et al. observed that for nanoparticles with a size ranging from 122 nm to 1000 nm in in vitro tests on the skin of a pig’s ear, NPs with a diameter of 643 nm were transported the furthest through the hair follicles [33]. Abdel-Hafez et al., using the confocal microscopy technique, proved that chitosan nanoparticles labeled with FITC applied to the surface of mouse skin penetrate deep into it [34]. The largest amount of CHNPs was observed in the hair follicles, which confirms that the follicular pathway plays the main role in the transdermal penetration of chitosan nanoparticles [31,34,35]. It is assumed that the obtained chitosan nanoparticles will be an excellent carrier of active substances using the skin route as a way of penetration. One of the goals of this research was to encapsulate the natural GBE extract in chitosan nanoparticles. Brown-colored Ch(GB)NPs were obtained, proving that the *Ginkgo biloba* extract was effectively sealed. In addition, FTIR-ATR analysis of the obtained Ch(Gb)NPs was performed. As a result of the deconvolution of the 1024 cm^−1^ and 1064 cm^−1^ peaks, the peaks (1) 1096, (2) 1066, (3) 1040 and (4) 1023 cm^−1^ were obtained, corresponding, respectively, to (1) PO_3_ groups stretching vibrations (symmetric and asymmetric) in TPP; (2) and (3) C–O stretches in GBE; and (4) C–O stretching vibration in chitosan (Figure 4) [3]. The results show that the GBE extract was successfully encapsulated in the chitosan nanoparticles. Encapsulation of plant extracts with natural polymers is an important sector of biomedicine, providing many advantages, i.e., an increased stability of natural active substances, distribution, an increased stability of drugs, prevention of the premature degradation of medicinal substances, which ensures greater bioactivity of nanoparticles and local therapy associated with low side effects [36]. The kinetics of active substance release from nanoparticles in two test media was carried out using a UV-Vis spectrophotometer. The release profile of the active substance from nanoparticles in an environment similar to physiological body fluids was assessed. The extract has only one barrier to overcome; therefore, the release of the extract at the beginning is greater (for both media), and in the water medium (pH 7.0), it stabilizes over time. In contrast, in saline (pH 5.8), the release over time increases at a slowed rate. This is due to the fact that GBE is first released from the periphery of the nanoparticles. GBE penetrates from the inside of the polymer matrix into the surrounding environment, and the polymer coating constitutes a diffusion barrier, hindering and slowing down the rate of penetration of the extract into the test medium. Based on the research on the kinetics of the release of the core material from Ch(GB)NPs, it can be concluded that in both cases, the release of the active substance takes place by passive diffusion. The mechanism of the plant extract release from ChNPs consists of (a) the release of active compounds bound in the surface layer of the polymer matrix of the nanoparticles; (b) diffusion by swelling of the matrix; and (c) the release of the extract as a result of polymer erosion, decomposition, hydrolysis or degradation of the NPs’ polymer scaffold, resulting in long-term drug release [37]. It has been confirmed that the release of the drug from ChNPs is characterized by a two-phase process, where initially, a very fast release occurs, followed by a slower, controlled release of the active compounds [37,38,39]. The release of the active substance from the polymer matrix is regulated by one of the following mechanisms: (a) the surface of the drug carrier is eroded, (b) breaking of the bonds on the surface or in the mass of the polymer or (c) diffusion of a closed extract. In many cases, the release of the extract is associated with the use of more than one of the above-mentioned points [40]. A kinetic analysis of the release of the active compounds (GBE) from the chitosan nanoparticles can be performed using mathematical models. The best fit of the release kinetics of GBE in the saline solution was obtained for the first-order model and for the release of GBE in water—for the Higuchi model. Slow-release kinetics due to diffusion are studied using zero-order, first-order and Higuchi models [37,41,42,43]. The slow and controlled release of the NPs’ extract is designed to maintain the dose of the active substance in the therapeutic range. Therefore, it is important to prepare polymeric drug delivery systems that have a zero-order drug release profile in which the active substance is released evenly [37,44]. Jonassen et al. have shown in their studies that chitosan has a rigid and extended conformation in water due to electrostatic repulsion between positively charged amino groups in chitosan chains. Charges are reduced when, instead of water, e.g., saline solution, is added as a medium. The screening effect in the saline medium reduces the electrostatic repulsion between the positively charged groups in the chitosan chains, and therefore, these chains are more flexible in a saline environment [45]. In addition, the pH of the environment has a key impact on the rate of release of the active substance from nanoparticles (water, pH 7.0; saline, pH 5.8). This confirms the results of our research related to the increased release of the *Ginkgo biloba* extract with Ch(GB)NPs in saline in relation to the aqueous medium.

The MTT assay is a sensitive, quantitative and reliable colorimetric test that evaluates the viability of a cell line and is based on the activity of mitochondrial dehydrogenase—LDH—in living cells associated with the reduction of the yellow, water-soluble substrate 3-(4,5-dimethylthiazol-2-yl)-2,5-diphenyltetrazolium bromide (MTT) into a deep blue/purple formazan product (insoluble in water). The cytotoxic effect was shown for the first time on different cell lines (HGF-1, PEA1 and HeLa). This study showed no cytotoxicity for normal cell line HGF-1 and a cytotoxic effect against the tested cancer line—PEA1—after treatment with pure GBE, ChNPs and Ch(GB)NPs. The GBE extract showed a strong cytotoxic effect for ovarian cancer in the tested concentration range (0–2.5 mg/mL) with IC_50_ = 0.335 mg/mL. A stronger cytotoxic effect against the tested cancer line was confirmed for the activity of Ch(GB)NPs with an IC_50_ = 0.178 mg/mL. In turn, for HeLa cancer cells, a weaker cytotoxic effect was demonstrated with IC_50_ = 0.975 mg/mL for the GBE extract and with IC_50_ = 0.505 mg/mL for the combination of the extract and nanoparticles (Ch(GB)NPs). This is the result of a synergistic effect between the bioactivity of chitosan nanoparticles and the *Ginkgo biloba* extract. The smallest cytotoxic effect was shown by the chitosan nanoparticles without the extract. An interesting aspect of this cytotoxicity assay is the lack of toxic effects of all the tested components against the normal human gingival (HGF-1) cell line. Other studies conducted by Kim et al. showed that GBE (EGb 761) had no cytotoxic effect on human fibroblasts at a concentration of 0.5 mg/mL [46]. Some components of EGb 761 have been shown to have anti-apoptotic properties [47]. On the other hand, Chao et al. demonstrated cytotoxic effect of GBE on human hepatocellular carcinoma cells (HepG2 and Hep3B) with IC_50_ = 0.75 mg/mL, in the range of concentrations tested (0–1.0 mg/mL) [47]. The cytotoxic effect of the GBE extract can be related to the necrosis and/or apoptosis that occurs. An increased necrosis indicator (LDH) release by GBE-treated cells indicates increased necrotic processes. In turn, an increased p53 protein expression suggests the activation of apoptotic pathways (although apoptosis markers have not been measured directly) [48]. Ginkgetin, which is an important component of EGb 761, at a concentration of 0.005 mg/mL, caused an increase in the level of hydrogen peroxide in the treated ovarian cancer cells and consequently led to the induction of apoptosis in OVCAR-3, where the main pathway was the activation of caspase as a result of hydrogen peroxide production and to a lesser extent, ginkgetin auto-oxidation [49]. Positively charged chitosan nanoparticles allow non-covalent binding to biological tissues and are used for drug delivery, which can be an alternative treatment method and help overcome the inadequacy of existing chemotherapy [50]. In addition, the literature reports that pure nanoparticles have an anticancer effect, which was confirmed in research conducted by Qi et al. They showed that ChNPs had a cytotoxic effect on liver (BEL7402), gastric (BGC823) and colon (Colo320) cancer cells at doses of IC_50_ = 0.015 mg/mL, IC_50_ = 0.021 mg/mL and IC_50_ = 0.028 mg/mL, respectively, in tested range concentrations of 0–0.1 mg/mL [51]. The same researchers also proved that chitosan nanoparticles do not have a cytotoxic effect on normal liver cells (L-02) in the same range of nanoparticle concentrations [51]. It has also been found that the particle size of nanoparticles plays a key role in the cytotoxic effect against cancer cells [51,52,53]. Very small nanoparticles showed greater accumulation into tumor cells and prolonged half-life in vivo due to the ability to avoid their uptake by the reticuloendothelial system [54]. Small nanoparticles can remain in circulation for a longer time, which in turn results in better bioavailability of the drug and an increased release of the drug contained in them in vitro [51].

A very important factor influencing the choice of chitosan as a matrix of nanoparticles is the possibility of their long-term persistence in the bloodstream. This allows for the delaying of their dissociation and effect in target cells, lesser side effects and high selectivity of action, e.g., on cancer cells [50].

## 4. Materials and Methods

### 4.1. Materials

The following materials were used: chitosan (Primex, Chitoclear fg 95, average MW~234.55 kDa, DD = 86.8%, 99.17 cP viscosity measured for 1% water solution in 20 °C, Siglufjordur, Iceland), acetic acid (Avantor, 80%, pure, Gliwice, Poland), Sodium tripolyphosphate pentabasic-TPP (Sigma-Aldrich-Merck, purum p.a., ≥98.0%, Cat. No.: 72061, Burlington, MA, USA), commercial medicinal product Tinctura Ginkgo bilobae, water-ethanol extract from the green leaves of *Ginkgo biloba* (concentrated to 10% solution to obtain nanoparticles and lyophilized for cytotoxicity studies, Phytopharm, Klęka, Poland), Polysorbate 80, Tween 80 (Lachner, s.r.o, Neratovice, Czech Republic), sodium chloride (Chempur, pure, Piekary Śląskie Poland) and distilled water.

### 4.2. Methods

#### 4.2.1. Preparation of Ch(GB)NPs

Ch(GB)NPs were obtained based on the ionotropic gelation method developed and described by Calvo et al. [55] and Porras-Gómez et al. [56] with a small modification. Firstly, chitosan was dissolved in a 0.5% *v/w* acid acetic solution to obtain a 0.5% *w/w* concentration. TPP was dissolved in distilled water to obtain a 0.5% *w/w* concentration. Tween 80 was dissolved in distilled water to obtain a 1.5% *w/w* concentration. A total of 1 mL of GBE was added into 10 mL of the chitosan solution, and then 4 mL of a 1.5% Tween 80 solution (as a surfactant) was added. Later, 2.5 mL of the TPP solution was added drop-wise to the chitosan and the GBE solution, and they were homogenized for 1 h. The final suspension of Ch(GB)NPs was centrifuged at 12,000 rpm for 20 min. Obtained nanoparticles were washed twice with water and centrifuged in the same conditions. Pure chitosan nanoparticles (ChNPs) were made according to the method described above, with the difference that instead of the GBE extract, the same amount of distilled water was added to the chitosan solution.

For biological studies (cytotoxicity on cell lines), the sample was washed twice with saline solution and centrifuged in the same conditions.

#### 4.2.2. Characterization of Ch(GB)NPs

##### The Size Analysis of Ch(GB)NPs

The average diameter and polydispersity index (PDI) of the obtained nanoparticles were measured by the Zeta Sizer Nano ZS (Malvern, UK) instrument. Measurements were made at 25 ℃ in a water medium. In order to determine the particle size and PDI values, 1 mL of the Ch(GB)NPs sample was placed in the ZEN0040 cuvette (Malvern, UK), and the results were analyzed using the dynamic light scattering (DLS) technique. In order to analyze the influence of nanoparticle aggregation on the size and polydispersion, measurements were carried out for nanoparticle solutions without preliminary sonification and after a 5 and 10 min preliminary sonification. Each sample was measured 3 × 11 times, and the results were analyzed by the Malvern Instruments Ltd. software.

##### The Morphology of Ch(GB)NPs

The nanoparticles’ morphology (the surface structure and shape) was evaluated using microphotography, obtained by means of a scanning electron microscope—SEM—in 12,000× and 120,000× magnification. For particles evaluated by SEM, the freeze-dried nanoparticles’ microcapsules were sprayed with gold and imaged on an FEI Quanta 200 model with the Q150R S vacuum sputtering machine (Hillsboro, OR, USA) to establish their morphological parameters.

#### 4.2.3. Encapsulation and Loading Efficiency

The efficiency of GBE encapsulation was determined by using the Perkin Elmer UV-Vis spectrophotometer (Waltham, MA, USA) with the Lambda 2 software (the indirect spectrophotometric method measuring absorbance in the range of spectra characteristic for rutin: 257–270 nm). The supernatant containing untrapped GBE (after separation from the centrifuged nanoparticles) was examined spectrophotometrically. Based on the absorbance value, the content of the untrapped GBE was determined (previously, the calibration curve was established). A formula was used to define the content of GBE loaded in the nanoparticles:

(1) $$\mathrm{EE}\%=\frac{{\left[C\right]}_{i}-{\left[C\right]}_{f}}{{\left[C\right]}_{i}}\times 100\%\;[50]$$

EE%—Encapsulation Efficiency, %

[C]_i_—Initial GBE concentration, mg/mL

[C]_f_—Concentration of untrapped GBE in supernatant, mg/mL

#### 4.2.4. The Rate of Release of GBE from Ch(GB)NPs

The same UV-Vis spectrophotometer with the software was used to study the kinetics of the core material release (GBE). The Ch(GB)NPs samples were prepared in the form of tablets for the study of release kinetics. A total of 0.05 g of each sample was pressed into a tablet with a diameter of 12 mm and poured over 5 mL of the test medium (the tests were carried out in water—pH 7.0—and saline—pH 5.8), based on the methodology presented by Siepmann et al. [57]. The flasks with the tested samples were closed tightly and placed in an incubator at 33 °C. Absorption studies were performed at the rutin wavelength in the range of 257–270 nm. Measurements were made at specified intervals by collecting 2 mL of the medium containing the above-mentioned samples, and the same volume of fresh medium was supplemented with the tested systems to the baseline volume.

Four different mathematical models (zero-order, first-order, Higuchi and the Korsmeyer–Peppas model) were fitted with the obtained cumulative drug release vs. time curves to describe the kinetics. To evaluate which model was followed, the value of the regression coefficient (R^2^) was determined and compared.

#### 4.2.5. FT-IR Analysis

Fourier transformed infrared spectroscopy (FT-IR) measurements were carried out using an FT-IR spectrophotometer (Nicolet iS50, Thermo Scientific, Waltham, MA, USA) in attenuated total reflection (ATR) mode. The analysis was performed in the measurement range of 4000–500 cm^−1^ with a resolution of 4.0 cm^−1^. The number of scans for the baseline and spectral acquisition was 32, and a DTGS ATR detector equipped with a diamond was used. Before performing the FTIR-ATR analysis, a background scan was performed, and the diamond crystal was cleaned with isopropyl alcohol to reduce interference. Three samples (chitosan powder, freeze-dried GBE and Ch(GB)NPs) were prepared for FTIR-ATR analysis in the same quantities. After the measurements, a graph of absorbance units as a function of the wavelength was prepared for each sample, and the characteristic peak associated with the chemical bond was defined.

#### 4.2.6. Cell Culture

The cancer cells PEA1, HeLa and normal HGF-1 cells (ATTC; Manassas, VA, USA) were grown and maintained in an appropriate medium, RPMI-1640, (EMEM and DMEM)/Ham’s F-12 50/50 mix, supplemented with 10% fetal bovine serum (FBS), glutamine (2 mM), penicillin (100 units/mL), and streptomycin (100 µg/mL) and additionally, 1% Non-Essential Amino Acids (NEAA) for HeLa. The cell cultures were grown in a carbon dioxide incubator (New Brunswick Galaxy^®®^ 170R CO_2_ Incubator) at 37 °C with 90% humidity and 5% CO_2_.

#### 4.2.7. MTT Assay for Cell Viability

The 3-(4,5-dimethylthiazol-2-yl)-2,5-diphenyltetrazolium bromide assay was used to measure cell viability in accordance with our earlier studies. Briefly, cells were cultured in 96-well plates and incubated with various concentrations of chitosan nanoparticles (ChNPs), the *Ginkgo biloba* extract and Ch(GB)NPs. After washing the cells twice with PBS, 100 μL of a medium containing MTT (0.5 mg/mL) was added to each well and the cells were incubated at 37 °C extra for 4 h. The medium was then removed so as not to disturb the formed formazan crystals. Next, 100 μL of DMSO was added to each well to dissolve the formazan crystals, and the absorbance of the dissolved blue formazan was read at 540 nm using a BioTek Synergy HT microplate reader (Bio-Tek Instruments, Winooski, VT, USA), with DMSO as a blank. The decrease in optical density by the compounds was used as a measurement of cell viability, normalized to cells incubated in the control medium, which were considered 100% viable.

## 5. Conclusions

As a result of this research, nanoparticles were obtained from a polymer of natural origin from renewable sources—chitosan. Inside, a natural *Ginkgo biloba* leaf extract was enclosed, which was confirmed by the results of the FTIR-ATR and UV-Vis tests (the release of GBE from the nanoparticles into the research medium: water and saline). The release into saline is much higher compared to water, which promises that the release of GBE from Ch(GB)NPs in the medium of physiological fluids under the conditions of use will be at a similar level. The size of the nanoparticles (confirmed by the analysis on the Zeta Sizer) gives a very good prognosis and creates the possibility of nanoparticles penetrating the follicular route through the skin, bypassing the stratum corneum, which is the barrier to penetration. These preliminary studies show, for the first time, the selectivity of the action of nanoparticles with the plant extract, no cytotoxic effect on normal line cells (HGF-1) and a visible cytotoxic effect on PEA1 and HeLa cancer cells. This preliminary research offers great opportunities and shows the potential to use nanotechnology solutions with the use of natural components in various branches of human and veterinary medicine.

In the future, the nanoparticles described in this manuscript will be encapsulated in larger structures, e.g., in microcapsules, and then immobilized into textile materials in order to obtain functional textiles. These materials will be used as a therapy supporting proper treatment by releasing active substances in the nanoparticles and absorbing them through the transfollicular route.

## Figures and Tables

**Table 1:** Particle size and polydispersity index of Ch(GB)NPs (with and without initial sonification for 5 and 10 min).

Sample	Sonification (min)	Size (nm)	PDI
Ch(GB)NPs	-	454.2	0.549
Ch(GB)NPs	5	374.9	0.512
Ch(GB)NPs	10	358.9	0.474

**Table 2:** Mathematical models and regression coefficient values of *Ginkgo biloba* extract release.

Drug Release Model	Medium—Water (Sample A)	Medium—Saline (Sample B)
Zero order	0.8298	0.8823
First order	0.8552	0.9104
Higuchi	0.9204	0.8586
Korsmeyer–Peppas	0.8608	0.8317

## Data Availability

Not applicable.

## Supplementary Materials

The following supporting information can be downloaded at: https://www.mdpi.com/article/10.3390/molecules28134950/s1, Figure S1. The rate of release of GBE from Ch(GB)NPs in water, pH 7.0 (A) and in saline, pH 5.8 (during the first hour of release); Figure S2: The rate of release of GBE from Ch(GB)NPs in water, pH 7.0 (A) and in saline, pH 5.8; Figure S3. The rate of release of GBE from Ch(GB)NPs in water, pH 7.0 (A) and in saline, pH 5.8 (without the correction); Figure S4: A plot of GBE release kinetics in water and in saline (Higuchi model); Figure S5: A plot of GBE release kinetics in water and in saline (First order model); Figure S6: A plot of GBE release kinetics in water and in saline (Zero order model); Figure S7: A plot of GBE release kinetics in water and in saline (Korsmeyer–Peppas model); Table S1: Data for the rate of release of GBE from Ch(GB)NPs in water and in saline; Table S2. Data for the rate of release of GBE from Ch(GB)NPs in water and in saline; Table S3: Data for Higuchi model of GBE release in water and in saline; Table S4: Data for First order model of GBE release in water and in saline; Table S5: Data for Zero order model of GBE release in water and in saline; Table S6: Data for Korsmeyer–Peppas model of GBE release in water and in saline.
