# Supplementary material for: Chitosan Nanoparticles-Preparation, Characterization and Their Combination with Ginkgo biloba Extract in Preliminary In Vitro Studies
Source: Molecules. 2023 Jun 23;28(13):4950. doi: 10.3390/molecules28134950 (PMC10343372; doi:10.3390/molecules28134950)

Table S1. Data for the rate of release of GBE from Ch(GB)NPs in water and in saline

	**Nano water**		**Nano saline**				
**czas**	**A**	**c, μg/ml**	**A**	**c, μg/ml**			
**5 min**	0.0286	9.53	0.1013	33.77	0.1013	5	1.6
**15 min**	0.0599	19.97	0.1270	42.33	0.1270	15	2.7
**30 min**	0.0761	25.37	0.2197	73.23	0.2197	30	3.4
**1h**	0.0768	25.60	0.2139	71.30	0.2139	60	4.1
**2h**	0.0776	25.87	0.2094	69.80	0.1894	120	4.8
**4h**	0.0842	28.07	0.2170	72.33	0.1970	240	5.5
**24h**	0.0930	31.00	0.2580	86.00	0.2580	1440	7.3
**96h**	0.1310	43.67	0.2930	97.67	0.2930	5760	8.6
**7d**	0.1500	50.00	0.3180	106.00	0.3180	10080	9.2
**14d**	0.1860	62.00	0.5480	182.67	0.5480	20160	9.9

Figure S1. The rate of release of GBE from Ch(GB)NPs in water, pH 7.0 (A) and in saline, pH 5.8 (during the first hour of release)

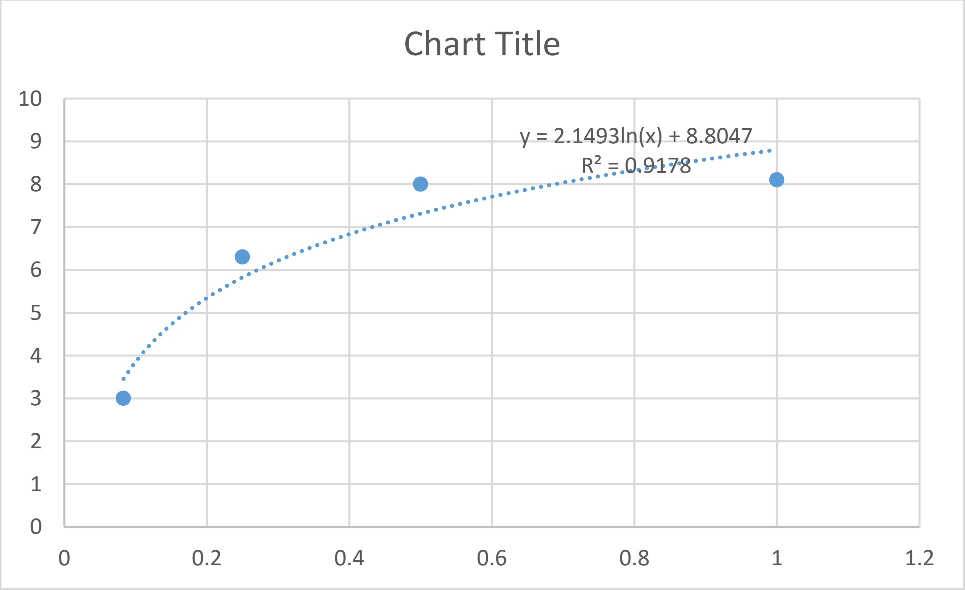

Table S2. Data for the rate of release of GBE from Ch(GB)NPs in water and in saline

time, h	concentration, μg/ml	time, h	concentration, μg/ml	
		0		
0.083	0.00953	0.083	0.03377	0.083
0.25	0.01997	0.25	0.04233	0.25
0.5	0.02537	0.5	0.07323	0.5
1	0.0266	1	0.0713	1
2	0.02733	2	0.07813	2
4	0.02973	4	0.08067	4
24	0.03067	24	0.086	
96	0.04367	96	0.09767	
168	0.05	168	0.12	

336	0.062	336	0.18267

Figure S2. The rate of release of GBE from Ch(GB)NPs in water, pH 7.0 (A) and in saline, pH 5.8

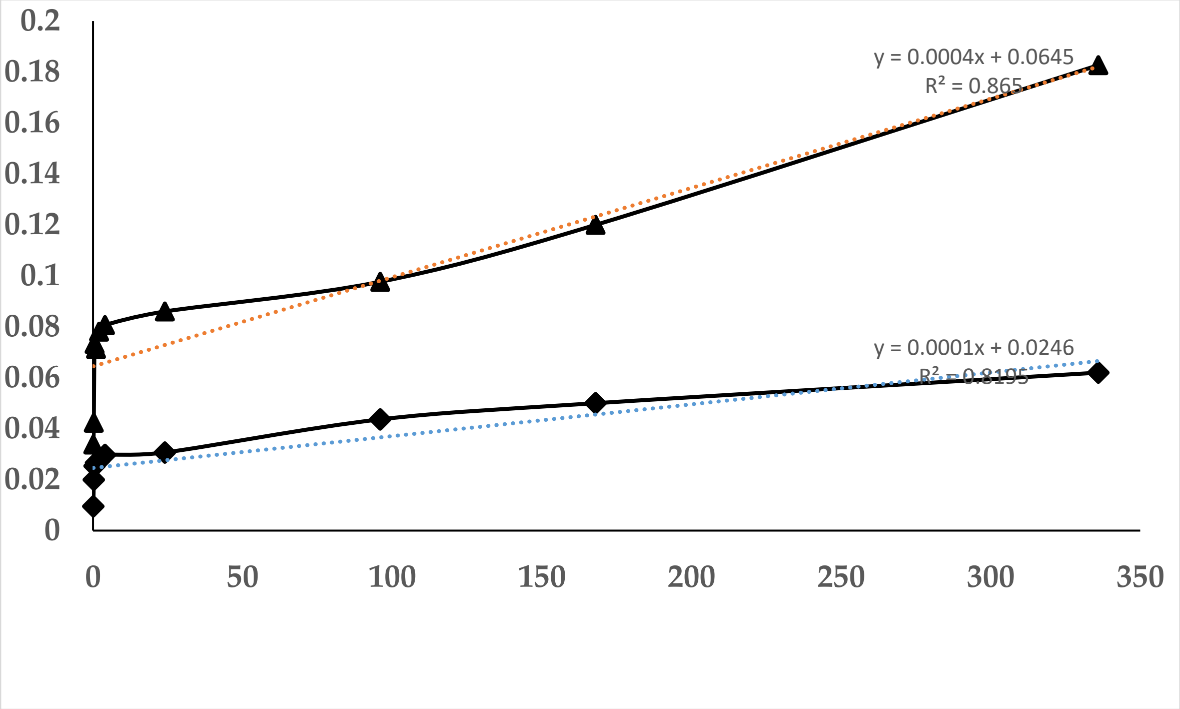

Figure S3. The rate of release of GBE from Ch(GB)NPs in water, pH 7.0 (A) and in saline, pH 5.8 (without the correction)

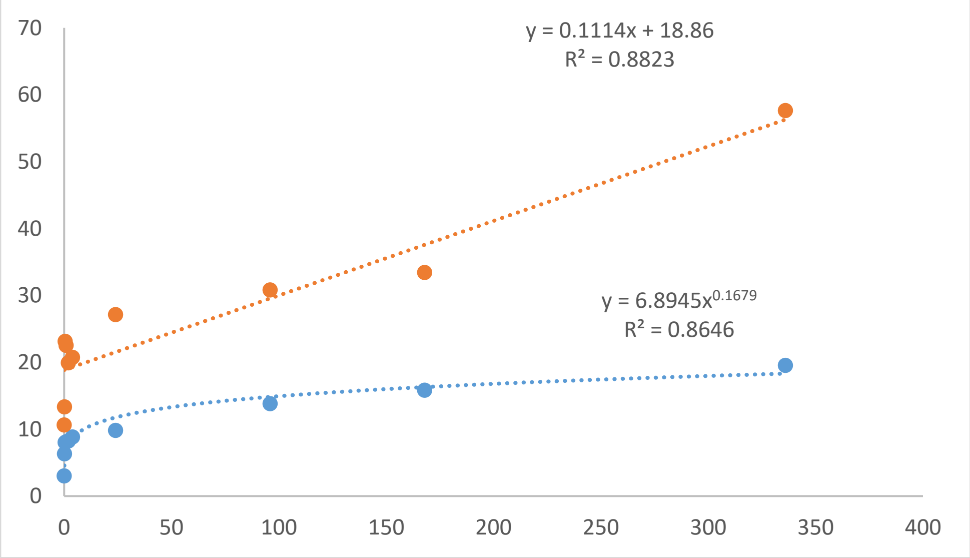

Table S3. Data for Higuchi model of GBE release in water and in saline

0.29	3	10.6
0.5	6.3	13.3
0.71	8	23.1
1	8.1	22.5
1.41	8.2	19.9
2	8.8	20.7

4.9	9.8	27.1
9.8	13.8	30.8
12.96	15.8	33.4
18.33	19.5	57.6

Figure S4. A plot of GBE release kinetics in water and in saline (Higuchi model)

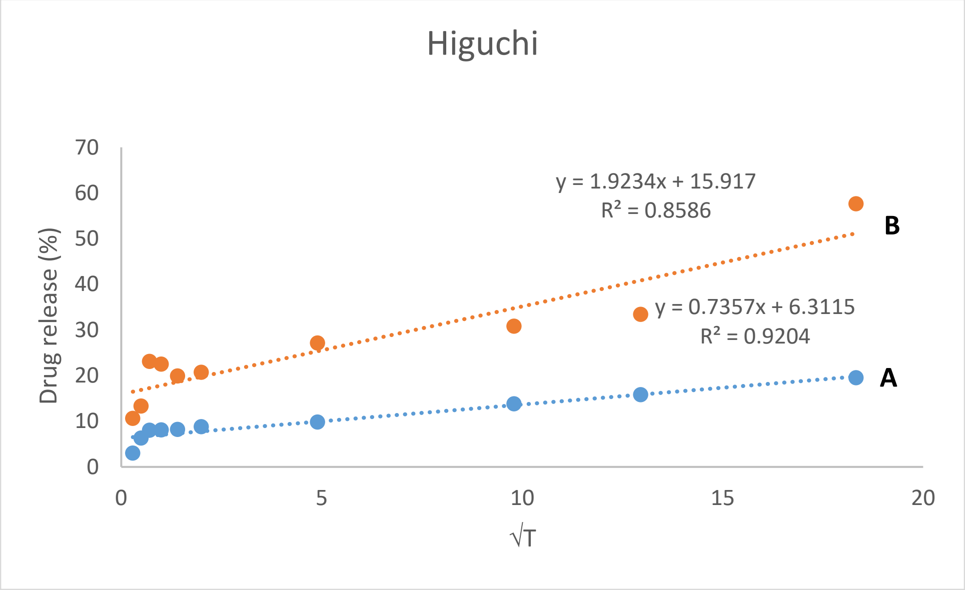

Table S4. Data for First order model of GBE release in water and in saline

0.083	-0.081	-0.163
0.25	-0.115	-0.194
0.5	-0.133	-0.313
1	-0.134	-0.305
2	-0.135	-0.272
4	-0.143	-0.282
24	-0.153	-0.367
96	-0.198	-0.418
168	-0.222	-0.457
336	-0.268	-0.909

Figure S5. A plot of GBE release kinetics in water and in saline (First order model)

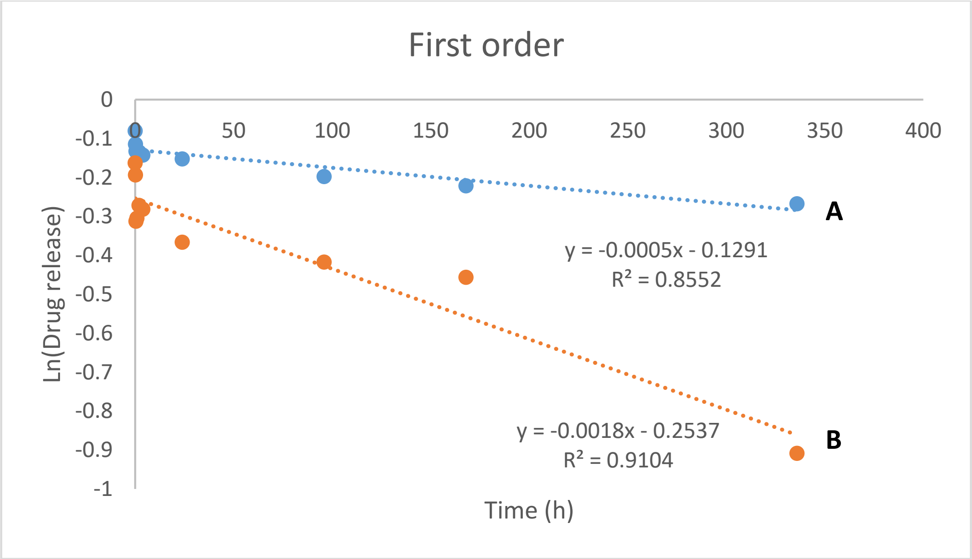

Table S5. Data for Zero order model of GBE release in water and in saline

0.083	3	10.6
0.25	6.3	13.3
0.5	8	23.1
1	8.1	22.5
2	8.2	19.9
4	8.8	20.7
24	9.8	27.1
96	13.8	30.8
168	15.8	33.4
336	19.5	57.6

Figure S6. A plot of GBE release kinetics in water and in saline (Zero order model)

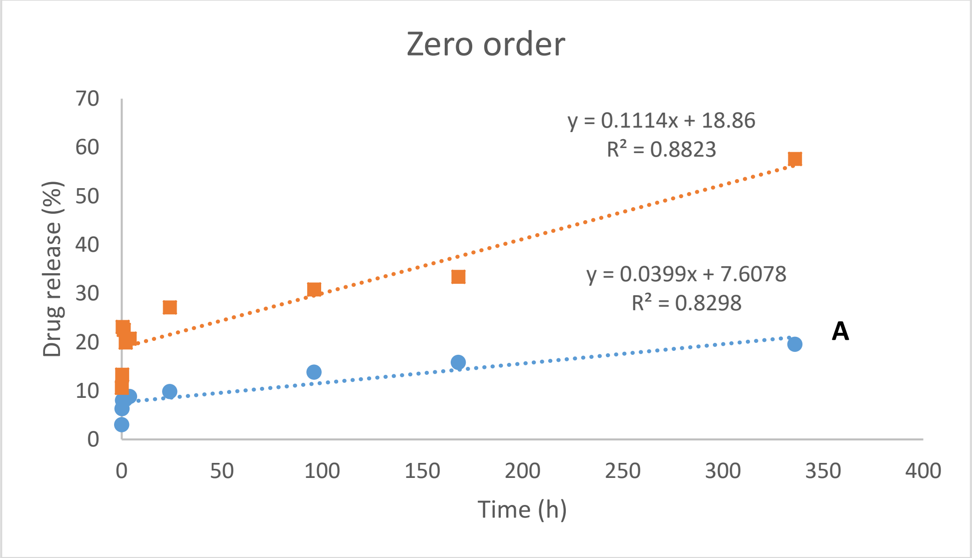

Table S6. Data for Korsmeyer-Peppas model of GBE release in water and in saline

1.6	-3.5	-2.24
2.7	-2.76	-2.01

3.4	-2.5	-1.46
4.1	-2.52	-1.49
4.8	-2.51	-1.61
5.5	-2.42	-1.57
7.3	-2.32	-1.3
8.6	-1.98	-1.18
9.2	-1.85	-1.09
9.9	-1.63	-0.55

Figure S7. A plot of GBE release kinetics in water and in saline (Korsmeyer-Peppas model)

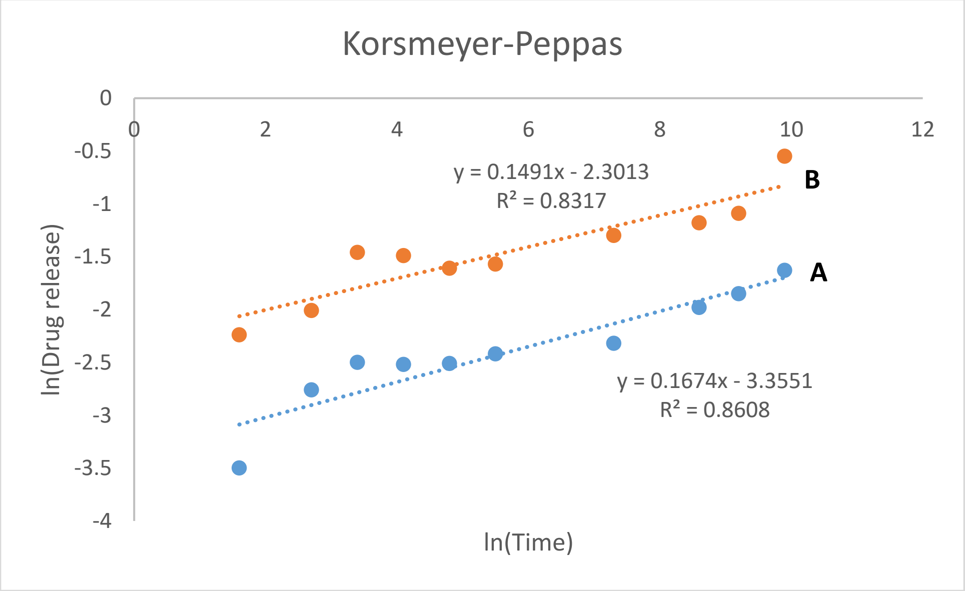

Supplement: Supplementary file 1 [file molecules-28-04950-s001.zip › molecules-2367981-supplementary.pdf]
